# Phytotoxicity and phytogenotoxicity of soil and air in the vicinity of a petrochemical plant in Płock (Poland)

**DOI:** 10.1007/s11356-020-08788-z

**Published:** 2020-04-18

**Authors:** Zbigniew M. Karaczun, Grażyna Obidoska, Barbara Żarska

**Affiliations:** Department of Environment Protection and Dendrology, Institute of Horticultural Sciences, Warsaw University of Life Sciences – SGGW, ul. Nowoursynowska 166, 02-787 Warsaw, Poland

**Keywords:** Toxicity, Genotoxicity, Bioindication, Phytotoxkit, Vicia RTA, TRAD MCN

## Abstract

Petrochemical industries have been widely recognised as important emission sources of airborne contaminants including heavy metals and polycyclic aromatic hydrocarbons PAHs, which affect the quality of air, soil and vegetation. In this study, our aim was to examine the phytotoxicity and phytogenotoxicity of soils and air in the vicinity of a petrochemical plant, in order to assess the potential threat of such industrial objects for crops and natural vegetation, in the cases when the allowable concentrations of contaminants are not exceeded and the plants in the vicinity visually do not seem to be affected. For phytotoxicity and phytogenotoxicity assessment, the Phytotoxkit and respectively Vicia RTA and TRAD MCN bioassays were used. According to our results, in spite of relatively low content of heavy metals and PAHs (hardly any exceedance of standards), the phytotoxicity and especially phytogenotoxicity of soil samples collected up to 18 km from the refinery were detected by the bioindicators. The phytogenotoxicity of air was also indicated within the distance of up to 12 km. We concluded that to obtain the complete view of the environmental risks in a surveyed area, a combination of chemical analysis of environmental samples with the bioindication methods should be implemented. In addition, setting the acceptable levels of contaminants should involve a more extensive use of bioindication methods (especially genotoxicity assessment).

## Introduction

Petrochemical industries have been widely recognised as important emission sources of airborne contaminants including heavy metals (Cortis et al. 2016; Bosco et al. 2005) and polycyclic aromatic hydrocarbons (PAHs) (Rao et al. 2008; Wang et al. 2017), which affect the quality of air (Augusto et al. 2010), soil (Nadal et al. 2007; Nadal et al. 2009; Li et al. 2009; Bayat et al. 2015), natural vegetation (Augusto et al. 2010; Bosco et al. 2005) and crops (Li et al. 2009) in the vicinity.

The refinery and petrochemical plant in Płock (Poland), which started operations in 1964, is still one of the largest units of this kind in Europe. It is located amidst agricultural land and valuable natural areas. As early as in the 1970s, it was clear that the plant had an adverse effect on the quality of the adjacent environment. Particularly vulnerable was the neighbouring soil due to very high emissions of particulate matter (PM) containing, among others, heavy metals and polycyclic aromatic hydrocarbons (PAHs) (Siewniak 1975). In the early 1990s, the plant introduced a number of remedies aimed at lowering the pollutant load released to the environment, which turned out to be quite successful (Żelazińska and Tuszewicki 2004). The emission to ambient air was significantly reduced and in subsequent years, thanks to further investments, it decreased even more (PKN Orlen 2004-7). The local soils fell into the category of not contaminated and showing the natural heavy metal content (according to current Polish standards: Regulation of the Minister of the Environment 2016). Also, in the samples of wheat grain and potato tubers, cultivated in the vicinity, only the natural content of heavy metals was observed (Karaczun et al. 2008). However, at the same time, preliminary investigations with selected plant bioindicators signalled phytotoxicity and phytogenotoxicity of soil samples collected in the area (Karaczun et al. 2008; Obidoska et al. 2006; Obidoska and Semenowicz 2009).

According to Greguskova and Micieta (2013), specific plant responses can be observed even at very low pollutants’ concentrations and some studies proved genotoxic effects of airborne pollutants emitted by a petrochemical plant on native plant species (Misik et al. 2007). It seems especially dangerous when meiotic abnormalities occur, leading to aberrant microspores, production of abortive pollen grains, and as a consequence, to seed failure. In certain species, the decreased reproductive success may lead to extinction (El Maataoui and Pichot 2001).

In this study, our aim was to examine the phytogenotoxicity of air in the vicinity of a petrochemical plant and phytotoxicity and phytogenotoxicity of soil samples collected from the area, in order to assess the potential threat of such industrial objects for crops and natural vegetation, in the cases when allowable concentrations of contaminants are not exceeded and the plants in the vicinity visually do not seem to be affected.

## Methods

### Study area and sampling sites

**Fig. 1:**
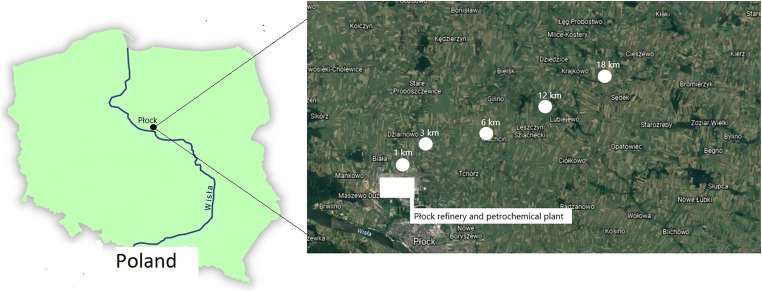
Location of research points, situated at 1–18-km distances in the north-eastern direction from Płock refinery and petrochemical plant

The research was conducted within an agricultural area located in the vicinity of the petrochemical plant in Płock. Soil samples were taken from the area lying in the north-eastern direction from the facility (main wind direction), at five research points at 1-km, 3-km, 6-km, 12-km and 18-km distance (Fig. 1). At the same sites, air testing was performed.

Each research point was located at a distance of at least 250 m from roads and other potential sources of emissions to the air. Five to 10 individual soil samples were collected from the stands with the surface of 7–10 m^2^ and mixed to form one representative sample of each research point. The samples were taken by using the Eagner’s sampler from the depth of 20 cm.

The arable soil layers, from which the samples were taken, had the following granulometric composition and pH in H_2_O: (1 km)—light clay sandy, 6.0; (3 km)—light clay sandy, 6.4; (6 km)—light clay sandy, 5.9; (12 km)—sand clayey solid sandy, 6.0; (18 km)—light clay sandy, 5.7.

### PAH content determination

Soil samples (20 g) were extracted with dichloromethane (CH_2_Cl_2_) in a fast extractor ASE200 at 100 °C and at pressure of 1500 psi. After purification and evaporation of the excess solvents, the samples concentrated in hexane to a volume of about 1 ml were subjected to gas chromatographic analysis with mass detection (GC-MS). The PAHs were determined using an Agilent device. It consisted of a gas chromatograph (type 6890 N), a mass detector (type 5973 N) and an autosampler (type 7683 B). The PAHs were separated according to the ISO 18287 on a capillary column type DB-5 MS + DG (J & W Scientific, USA) with a constant helium flow as carrier gas.

Quantitative and qualitative analyses of PAHs were carried out in the SIM (Selected Ion Monitoring) mass spectrometer mode. Identification of individual compounds was based on the analysis of characteristic ions and confirmatory ions in accordance with ISO 18287. The quality of the determinations was subject to control which included the analysis of the blank sample and analysis of the laboratory reference material. PAH content in soil samples was made in duplicate. The results given in Table 1 do not include a correction for recovery. For the applied method, the precision was 2–6%, the correctness ranged from 65 to 90%, MDL (Method of Detection Limit) for individual PAHs from 0.02 to 0.84 μg/kg and MQL (Method Quantification Limit)—0.05–2.53 μg/kg.

**Table 1:** PAH content in soils at 1–18-km distances from the petrochemical plant in Płock (μg/kg d.m.)

Distance						
	MQL* (μg/kg)	1 km	3 km	6 km	12 km	18 km
Means ±SD						
Naphthalene	1.9	11.6 ± 0.57	10.4 ± 0.85	8.0 ± 0	12.4 ± 3.75	8.0 ± 0.04
Acenaphthylene	0.2	2.8 ± 1.13	2.2 ± 0.21	2.2 ± 0.28	2.5 ± 0.71	2.2 ± 0.28
Acenaphthene	2.5	6.4 ± 2.26	5.6 ± 3.39	5.7 ± 3.32	5.6 ± 3.39	4.6 ± 1.98
Fluorene	2.3	7.25 ± 0.35	5.3 ± 0.99	5.4 ± 0.85	5.4 ± 0.85	4.7 ± 0.92
Phenanthrene	1.2	45.5 ± 6.36	31.8 ± 7.35	31.1 ± 4.10	29.5 ± 9.26	27.6 ± 6.29
Anthracene	1.2	5.2 ± 1.7	4.1 ± 1.34	3.9 ± 1.56	3.75 ± 0.35	4.0 ± 1.48
Fluoranthene	0.5	67.5 ± 4.95	26.5 ± 5.02	21.6 ± 3.46	26.2 ± 3.11	24.1 ± 2.76
Pyrene	0.3	56.0 ± 2.83	22.2 ± 4.03	18.3 ± 3.8	23.1 ± 4.31	19.9 ± 3.04
Benzo (a) anthracene	0.1	32.5 ± 3.54	12.5 ± 3.54	11.6 ± 3.39	13.3 ± 3.18	11.5 ± 3.61
Chrysene	0.1	39.5 ± 0.71	16.5 ± 2.12	13.0 ± 2.83	16.6 ± 3.68	14.2 ± 1.20
Benzo (b) fluoranthene	0.3	44.0 ± 14.14	38.9 ± 8.57	20.6 ± 3.39	27.1 ± 7.21	25.3 ± 1.06
Benzo (k) fluoranthene	0.2	32.0 ± 16.97	19.8 ± 3.11	10.2 ± 2.55	15.1 ± 4.38	8.3 ± 2.12
Benzo (a) pyrene	0.2	26.0 ± 15.56	20.7 ± 8.91	13.7 ± 3.25	16.5 ± 3.46	14.8 ± 1.77
Indeno (1,2,3-cd) pyrene	0.5	29.0 ± 1.41	36.5 ± 12.09	18.5 ± 3.54	21.4 ± 4.81	17.8 ± 4.53
Dibenzo (a,. h) anthracene	1.0	9.0 ± 4.24	8.8 ± 3.11	7.7 ± 1.84	9.7 ± 0.99	7.8 ± 1.70
Benzo (ghi) perylene	1.1	25.5 ± 4.95	26.0 ± 7.07	13.7 ± 3.25	15.9 ± 2.69	14.5 ± 2.12
Σ 16 PAH	5.3	439.8 ± 5.30	287.6 ± 71.29	205.1 ± 40.87	243.8 ± 20.93	208.8 ± 32.53

*Limit of determination for the method

### Heavy metal content determination

For the extraction of metals from soil samples, accelerated mineralisation under pressure in the Mars Xpress apparatus (CEM Company) was used. Air dry soil samples (0.5 g) were mineralised with the addition of 10 ml of royal water. Quantitative analysis of metals was carried out using an ICP-MS device (type 7500 Series, AGILENT Company). For the excitation of the plasma and as the carrier gas, argon was used and as reaction/collisional gases, hydrogen and helium. The limit of quantification of the method (MQL) for metals was on average 0.01–0.02 mg kg^−1^, and the accuracy was 10%.

### Phytotoxicity and phytogenotoxicity bioindication

Phytotoxicity of soils was tested using the standard rapid phytotoxicity microbiotest Phytotoxkit – MicroBioTests Inc., Belgium (PHYTOTOXKIT 2004) with three test species: *Lepidium sativum*, *Sinapis alba* and *Triticum aestivum.* Soil samples collected at 1–18-km distances from refinery were dried, sieved, placed in Phytotox test plates, moistened with tap water up to total water capacity level, covered with black paper filters and left for 1 h. Subsequently 15 seeds were sown per each plate, on the soaked paper filter. Rinsed river sand was used as the control (reference) sample. The plates were incubated in the dark, 25 °C, for 3 days. The reaction of the test plants to soil samples was assessed in reference to control using two parameters: seed germination rate and seedling root elongation. The experiment was set in 6 replications.

For soil phytogenotoxicity assessment, Vicia root tip assay (Vicia RTA) was performed. *Vicia faba var. minor* seeds germinated and produced primary roots in Petri dishes filled with paper filters soaked with tap water. Ready bioindicators, primary rooted seedlings, were subsequently placed in soil samples and in rinsed sand for control. The exposure lasted 5 days. For observations, soil-grown secondary roots were used (3 replications for each distance). After collection, they were fixed in Carnoy solution (1:3 glacial acetic acid and 96% ethanol) and stored in 70% ethanol. For slide preparation, the roots were hydrolysed in 1 M HCl solution (room temperature, 20 min) and stained with 2% aceto-orcein. The slides were analysed under optical microscope in × 400 magnification. In each of them, the percentages of anaphase and telophase aberrations (AAT) such as bridges, vagrant chromosomes or fragments were evaluated. Also, mitotic indices (MI) were calculated in 1000 cells and phase indices (PhI) in 200 dividing cells.

$$ \mathrm{MI}=M/N\times 100 $$

*M*—number of dividing cells (in any of phases of mitosis); *N*—total number of cells (min. 1000).

$$ \mathrm{PhI}=P/M\times 100 $$

*P*—number of cells in a certain phase of mitosis; *M*—number of dividing cells (min. 200).

Air phytogenotoxicity was assessed with TRAD MCN assay performed in accordance with the slightly modified protocol by Ma et al. (1994). Inflorescences of *Tradescantia hirsutiflora x subacaulis* clone 4430 were exposed for 8 h to ambient air (day time, 20–23 °C) at 1–18-km distances from the refinery and the control ones to clean ambient air in the Natura 2000 site in Puszcza Biała. After collection, they were fixed in Carnoy solution (24 h) and stored in 70% ethanol. Isolated anthers were squeezed and stained with 2% aceto-orcein. For scoring of micronuclei (MCN), × 400 magnification was used and 3000 tetrads per each distance (3 × 1000) were observed.

The results, presented as means ± standard deviations, were subjected to one-way analysis of variance (ANOVA) and Tukey post hoc test, with statistical significance level set at *p* = 0.05. Selected correlation coefficients’ significances were also tested (Statistica 13.3).

## Results and discussion

### PAH and metal content in soils

The concentrations of 16 PAHs and total PAH content as well as four heavy metals being the most typical petrochemical contaminants: Cd, Pb, Cr and Zn (Nadal et al. 2007; Nadal et al. 2009; Li et al. 2009) are given in Tables 1 and 2. The total PAH content at 1–18-km distances from the Płock refinery was, according to a 6-degree (0–5) scale (IUNG 2017), classified as class 1—indicating just an increased content in comparison with the natural one. It was highest at 1 km, dropping down with the growing distance (Table 1). Similarly, highest particulate matter and PAH contaminations were observed in the closest vicinity of the refinery (0.5 km), decreasing with the growing distance by Rao et al. (2008).

**Table 2:** Heavy metal content in soils at 1–18-km distances from the petrochemical plant in Płock (mg/kg d.m.)

Distance						
	MQL* (mg/kg)	1 km	3 km	6 km	12 km	18 km
Means ±SD						
Pb	0.02	67.2 ± 12.66	98.7 ± 25.88	136.0 ± 31.11	89.3 ± 12.30	72.8 ± 31.47
Cr	0.01	4.0 ± 3.11	5.7 ± 4.88	7.3 ± 6.83	5.5 ± 5.37	4.3 ± 3.75
Zn	0.01	32.65 ± 12.23	24.55 ± 2.05	31.80 ± 5.37	24.5 ± 3.54	21.75 ± 1.06
Cd	0.01	0.55 ± 0.06	0.76 ± 0.37	0.95 ± 0.63	0.75 ± 0.35	0.74 ± 0.33

*Limit of determination for the method

The concentration of heavy metals in the examined soils was rather low (Table 2) and did not exceed the Polish standards for agricultural light, mineral soils, except for lead content which at the distance of 6 km exceeded the standard by 36 mg/kg d.m. (Regulation of the Minister of the Environment 2016).

### Phytotoxicity and phytogenotoxicity of soils

**Fig. 2:**
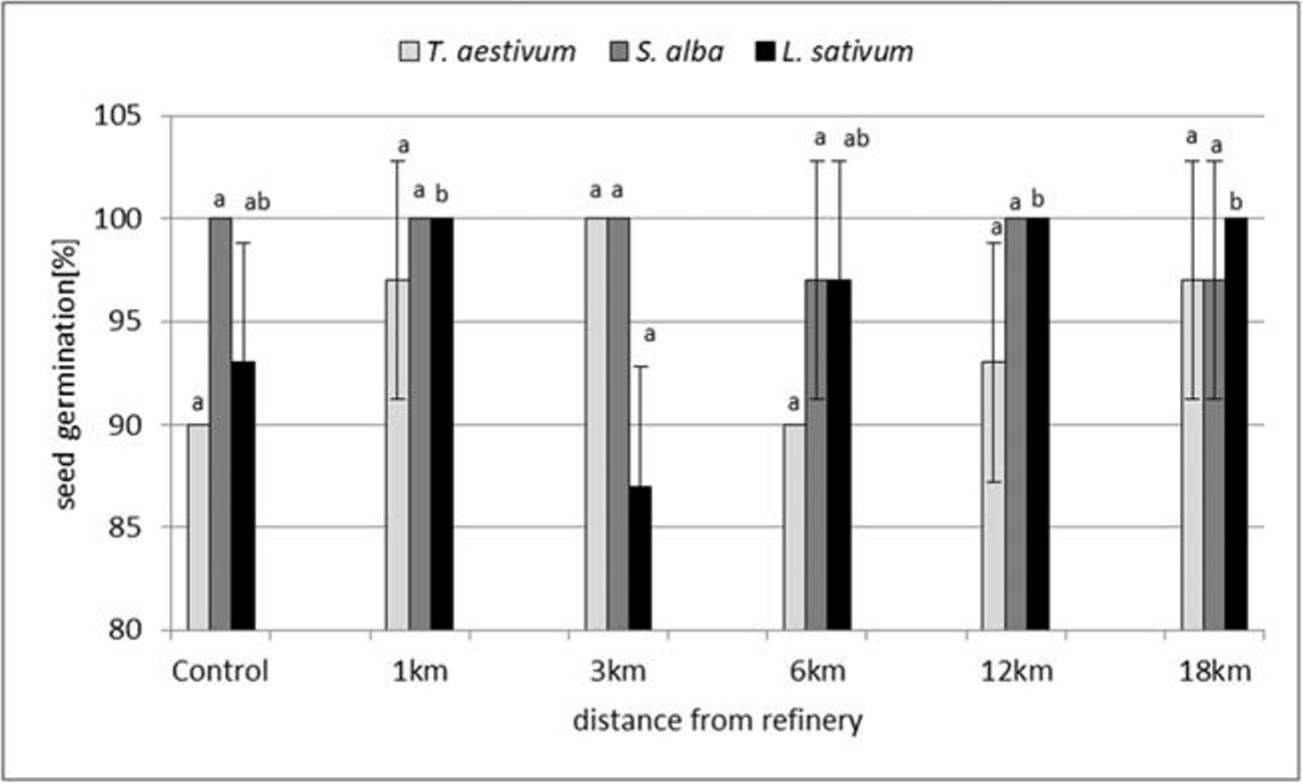
Seed germination of test species in soils taken at 1–18-km distances from the Płock refinery. The means marked with the same letter within one species do not differ significantly (*p* = 0.05)

**Fig. 3:**
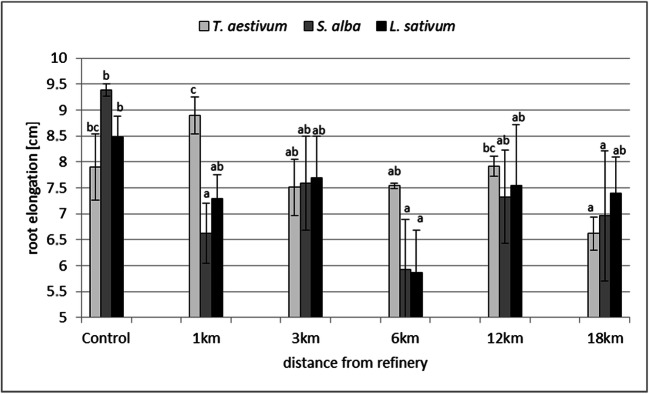
Root elongation of test species in soils taken at 1–18-km distances from the Płock refinery. The means marked with the same letter within one species do not differ significantly (*p* = 0.05)

Seed germination in *L. sativum*, *S. alba* and *T. aestivum* did not indicate soil phytotoxicity at any distance from petrochemical plant (Fig. 2). However, root elongation, which is a more sensitive phytotoxicity testing parameter (An 2004), was affected in all of the test species, although there were differences in responses. The most significant effect was observed in *S. alba*: the phytotoxicity was observed in soil samples taken from 1-km, 6-km and 18-km distances from the petrochemical plant*.* At 6-km distance, it was confirmed in *L. sativum* and at 18-km distance in *T. aestivum* (Fig. 3).

The phytotoxicity of soil samples in 1–18-km distances from the same refinery was previously reported on the basis of pot experiments (seedling emergence and growth test) (Karaczun et al. 2008; Obidoska and Semenowicz 2009), indicating the highest level at 1-, 6- and 18-km distances (Karaczun et al. 2008). The short-term Phytotox test is less sensitive than a pot experiment (shorter duration, limited contact of roots with tested soil covered by paper filters), but it seems to confirm soil phytotoxicity at 1-, 6- and 18-km distances from the refinery.

For phytogenotoxicity testing of soil samples, the recommended (Cotelle et al. 2015) *Vicia faba* bioindicator was applied. Firstly, the parameters of mitosis performance were calculated. In comparison with control, no inhibition in root meristematic cell divisions was observed at soil samples from any of the distances. The mitotic index (MI) in control was higher than in the tested soils, but the differences were not statistically significant. The phase indices (PhI), except for telophase, were not different from the control either (Table 3).

**Table 3:** Mitotic index (MI) and phase indices (PhI) in *Vicia faba* roots placed in soils taken at 1–18-km distances from the Płock refinery

Distance						
	Control	1 km	3 km	6 km	12 km	18 km
Means ±SD						
Mitotic index	10.6 ± 1.1a	8.7 ± 0.7a	8.9 ± 0.7a	9.9 ± 1.7a	7.8 ± 1.3a	8.5 ± 0.6a
Phase indices:						
Prophase	63.5 ± 1.7a	69.1 ± 2.6a	69.4 ± 1.2a	64.4 ± 4.8a	59.8 ± 3.0a	62.7 ± 6.3a
Metaphase	13.4 ± 2.2a	11.5 ± 2.3a	12.1 ± 2.7a	15.5 ± 3.2a	15.1 ± 3.0a	15.6 ± 1.5a
Anaphase	6.5 ± 2.7a	6.6 ± 2.0a	6.9 ± 0.2a	7.1 ± 1.6a	9.1 ± 0.3a	9.3 ± 4.2a
Telophase	16.9 ± 1.8a	12.5 ± 1.3c	11.5 ± 1.5c	13.1 ± 0.4bc	16.1 ± 0.7ab	12.4 ± 1.3c

The row means marked with the same letter do not differ significantly (*p* = 0.05)

**Fig. 4:**
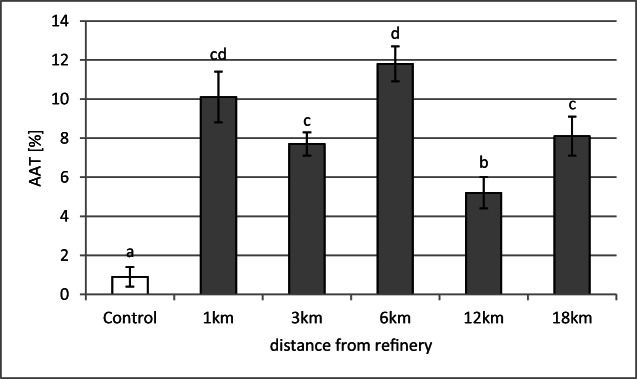
Frequency of anaphase and telophase aberrations (AAT) in *Vicia faba* roots placed in soils taken at 1–18-km distances from the Płock refinery. The means marked with the same letter do not differ significantly (*p* = 0.05)

Observations of anaphases and telophases, however, revealed a significant number of aberrations (AAT), such as chromosomal bridges, vagrant chromosomes and fragments of chromosomes. We concluded that the soil samples from each of the distances up to 18 km showed genotoxic effects on meristematic cells of *Vicia faba* roots (Fig. 4); especially high at 1- and 6-km distances (no statistically significant difference between the two mentioned samples), dropping significantly at 3, 12 and 18 km (in comparison with the highest score at 6 km).

### Phytogenotoxicity of air

**Fig. 5:**
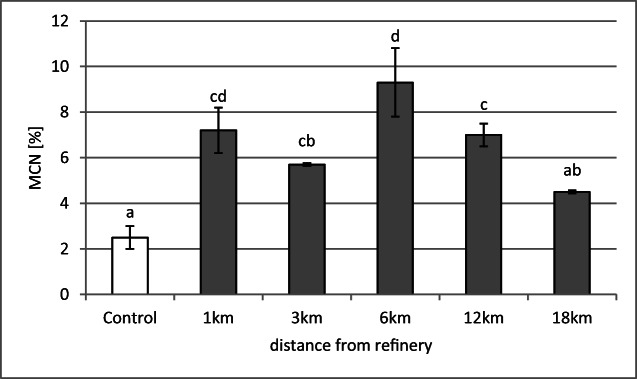
Frequency of micronuclei (MCN) in *Tradescantia* exposed to air at 1–18-km distances from the Płock refinery. The means marked with the same letter do not differ significantly (*p* = 0.05)

Phytogenotoxicity assessment of ambient air with TRAD MCN assay indicated the presence of phytogenotoxic factors up to 12-km distances from the refinery. For 18-km distance, the result, in comparison with control, was not statistically significant. The pattern was quite similar to soil phytogenotoxicity in the examined distances: the highest score of micronuclei in *Tradescantia* tetrads (%MCN) was noted for the inflorescences exposed at the 6-km distance, not significantly lower at 1 km, and a statistically significant drop in comparison with the highest score (6 km) at 3- and 12-km distances (Fig. 5).

### Correlation between specific pollutants and test plant responses

Statistically significant negative correlations were observed between lead and cadmium contents in soils and the root elongation of *L. sativum* (Table 4).

**Table 4:** Correlation between specific pollutants and test plants responses

	Pb	Cd	PAH
AAT (*V. faba*)	0.48	0.30	0.21
MCN (*T. hirsutiflora x subacaulis*)	0.55*	0.26	0.06
Root elongation (*L. sativum*)	− 0.73*	− 0.67*	0.07

*Correlation coefficient is significant (*p* = 0.05)

In case of one of the phytogenotoxicity parameters—AAT in *V. faba* roots and the contaminants of soil: lead, cadmium and PAH, the correlation coefficient was not statistically significant. Nevertheless, there was a linear positive relationship between MCN formation in *Tradescantia* and lead content in soils.

Because the investigated area is of agricultural type, distanced from other local sources of pollution, the petrochemical plant is highly suspected of being the main source of bioindicated phytotoxic and phytogenotoxic agents. Similar methods have already detected the presence of toxic and genotoxic airborne pollutants in the vicinity of other petrochemical plants (Cortis et al. 2016; Misik et al. 2007) and the relation between genotoxicity and the distance from such source was also noted (Greguskova and Micieta 2013; Rao et al. 2008). Moreover, our results are consistent with previous observations indicating that pollution forms two concentric zones around the petrochemical plant in Płock: first in the nearest vicinity (1 km) and second at the distance of 5–6 km. For the first time, the occurrence of such areas was signalled in 1970s (Biernacka et al. 1983) and subsequently confirmed in the 1980s (Karaczun and Indeka 1995; Karaczun 1995; Nowicki 1985). This supports the thesis of the plant being the source of detected phytotoxic and phytogenotoxic factors in the investigated area.

The highest phytogenotoxicity of soils was observed at the distances of 1 and 6 km from the refinery. In 1-km distance, the elevated total content of deposited airborne PAHs may be the reason (Table 1). The concentration was especially increased for low and intermediate molecular weight PAHs (3–4 rings), such as phenanthrene, fluoranthene, pyrene, benzo(a)anthracene and chrysene, which may quite easily be uptaken by roots (Kipopoulou et al. 1999) and possibly cause adverse effects. Phytotoxicity (Somtrakoon and Chouychai 2013) and genotoxicity (Ramos de Rainho et al. 2013) of PAHs have been reported previously. In soils located at 6-km distance, however, the total content of PAHs was about 50% lower than at 1 km and very similar to the content in the samples taken from 12 to 18 km. Instead, the contamination with lead was exceeding the acceptable level set for light, mineral agricultural soils (Regulation of the Minister of the Environment 2016). Lead has previously been reported to affect very strongly seed germination and root elongation of seedlings (Cavusoglu et al. 2010; Sethy and Gosh 2013) and to be genotoxic to *Vicia faba* roots (Shahid et al. 2011) or *Tradescantia* pollen mother cells (Patra et al. 2004).

Before the contaminants were deposited and accumulated in soils, they were emitted to the air and transported in this medium with the wind. TRAD MCN assay indicated phytogenotoxicity of air in the examined area up to 12 km from the refinery. It was positively correlated with lead content in soil. According to Misik et al. (2007), the results of genotoxicity assessment in the vicinity of a petrochemical plant obtained using the TRAD MCN assay and the pollen abortion assay in native species (*Chelidonium majus*, *Clematis vitalba*, *Cichorium intybus*, *Linaria vulgaris*) were coherent. Similar observations in contaminated areas were reported also by other authors (Solenska et al. 2006; Greguskova and Micieta 2013). In our study, we did not examine the native species, but in *Tradescantia* tetrads, micronuclei did appear, which suggests that in wild plants and crops, abnormal microspores, leading to the production of abortive pollen grains, could have appeared as well. It should be highlighted again, that pollination is crucial for seed production and reproductive success of plants. Pollen limitation especially affects annuals, because their persistence mostly depends on seeds (Pflugshaupt et al. 2002). High percentage of abortive fruits with empty seeds my even lead to the extinction of some sensitive species (Pflugshaupt et al. 2002; El Maataoui and Pichot 2001) and may cause economic loss in the case of crops.

Despite the fact that the results of chemical analysis of soil contamination in the petrochemical plant vicinity were not alerting (according to current standards), bioindication did detect the presence of phytotoxic and phytogenotoxic factors, potentially harmful to native vegetation and crops. This confirms the statement of Greguskova and Micieta (2013) that even at very low concentrations of pollutants, specific plant responses can be observed. It should be thus recommended to use chemical analysis combined with bioindication methods to obtain the complete view of the environmental risks in a surveyed area. It also seems that when setting the acceptable levels of contaminants, bioindication methods (especially genotoxicity assessment) should be applied to a greater extent and perhaps the current standards should be reconsidered in the future.

## Conclusions

1. In spite of a relatively low (according to current standards) contamination of soils with heavy metals and PAHs in the vicinity of a petrochemical plant, phytotoxicity and especially phytogenotoxicity of soil samples and air were detected by bioindicators.
2. Because the bioindicators’ responses were observed at low contaminants’ concentrations, it is recommended to combine chemical analysis of environmental samples with bioindication methods to obtain the complete view of the environmental risks in a surveyed area.
3. It seems that when setting the acceptable levels of contaminants, bioindication methods (especially genotoxicity assessment) should be applied to a greater extent, and the current standards should be reconsidered in the future.
